# Local Translation in Nervous System Pathologies

**DOI:** 10.3389/fnint.2021.689208

**Published:** 2021-06-29

**Authors:** María Gamarra, Aida de la Cruz, Maite Blanco-Urrejola, Jimena Baleriola

**Affiliations:** ^1^Laboratory of Local Translation in Neurons and Glia, Achucarro Basque Center for Neuroscience, Leioa, Spain; ^2^Departamento de Neurociencias, Universidad del País Vasco (UPV/EHU), Leioa, Spain; ^3^Departamento de Biología Celular e Histología, Universidad del País Vasco (UPV/EHU), Leioa, Spain; ^4^Ikerbasque, Basque Foundation for Science, Bilbao, Spain

**Keywords:** local translation, RNA localization, RNA binding proteins, mRNA transport, nervous system pathologies

## Abstract

Dendrites and axons can extend dozens to hundreds of centimeters away from the cell body so that a single neuron can sense and respond to thousands of stimuli. Thus, for an accurate function of dendrites and axons the neuronal proteome needs to be asymmetrically distributed within neurons. Protein asymmetry can be achieved by the transport of the protein itself or the transport of the mRNA that is then translated at target sites in neuronal processes. The latter transport mechanism implies local translation of localized mRNAs. The role of local translation in nervous system (NS) development and maintenance is well established, but recently there is growing evidence that this mechanism and its deregulation are also relevant in NS pathologies, including neurodegenerative diseases. For instance, upon pathological signals disease-related proteins can be locally synthesized in dendrites and axons. Locally synthesized proteins can exert their effects at or close to the site of translation, or they can be delivered to distal compartments like the nucleus and induce transcriptional responses that lead to neurodegeneration, nerve regeneration and other cell-wide responses. Relevant key players in the process of local protein synthesis are RNA binding proteins (RBPs), responsible for mRNA transport to neurites. Several neurological and neurodegenerative disorders, including amyotrophic lateral sclerosis or spinal motor atrophy, are characterized by mutations in genes encoding for RBPs and consequently mRNA localization and local translation are impaired. In other diseases changes in the local mRNA repertoire and altered local protein synthesis have been reported. In this review, we will discuss how deregulation of localized translation at different levels can contribute to the development and progression of nervous system pathologies.

## Introduction

**FIGURE 1:**
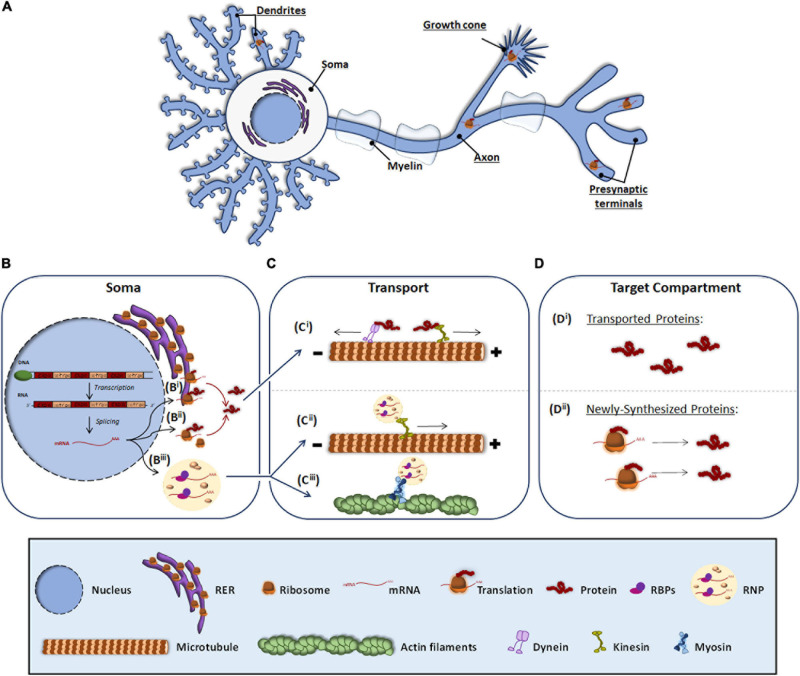
Protein and RNA transport to peripheral subneuronal compartments. Roughly it is underlined the neuronal compartments where local protein synthesis occurs **(A)**. In the soma, expressly in the nucleus, DNA is transcribed to RNA and after splicing, the resulting mRNA **(B)** can be either processed by the classical somatic translation or the local protein synthesis. For the classical one, the mRNA is translated by ribosomes associated to RER **(B^i^)** and free cytosolic ribosomes **(B^ii^)**. Synthetized proteins are transported **(C)** through dyneins or kinesis associated to microtubules **(C^i^)** to target compartments where elicit their function **(D^i^)**. For local translation, mRNA is associated to RBPs and assembled as RNP granules **(B^iii^)**, which can be transported through kinesis **(C^ii^)** or by myosins linked to actin filaments **(C^iii^)**. At the target compartments **(D)**, transported mRNAs are locally translated, and the newly synthesized proteins **(D^ii^)** elicit their function.

Neurons are morphologically the most complex cells in the nervous system (NS). They consist of a soma, from which several processes emerge. The characteristic asymmetric morphology of neurons allows them to create stable neural circuits. Through their dendrites and axons, neuronal processes are capable of receiving and integrating incoming signals from presynaptic terminals and transmitting them through signaling pathways to give response to diverse stimuli. The structural and functional asymmetry of neurons requires the asymmetrical distribution of the neuronal proteome, which is achieved by the transport of mature proteins or the transport of transcripts to target compartments in neuronal processes. In this article, we focus on the latter mechanism by which localized messenger RNAs (mRNAs) are transformed into proteins through a process known as local translation or local protein synthesis (Rangaraju et al., 2017; Holt et al., 2019) (Figure 1A).

## Local Translation for Neuronal Homeostasis

Protein synthesis is an essential mechanism to ensure proper cell homeostasis. Thereby, RNA translation and protein transport to subneuronal domains have been the focus of particular concern for many researchers. Although for years it was thought that protein synthesis takes place only in the soma, it is now accepted that the transport of proteins from the soma to target subcellular regions is not the only way to supply proteins to distal neuronal processes (Alberts et al., 2002). mRNAs can also be transported to subcellular domains for their subsequent translation into proteins. This phenomenon is known as local protein synthesis (Holt et al., 2019). Taking into consideration that in some vertebrates dendrites can extend up to a dozen millimeters from the soma and that axons can measure more than one meter (Bannister and Larkman, 1995) it is not surprising that local protein synthesis serves as a means for neuronal processes to rapidly react to diverse environmental stimuli. This translation mechanism was first described in 1960 when local synthesis of acetylcholinesterase in axons was detected using isotopically labeled amino acids (Koenig and Koelle, 1960). Subsequently, some other studies were carried out in different species: local protein synthesis was also observed in isolated axons of the giant squid (Giuditta et al., 1968) and nerve fibers in goldfish (Edstrom and Sjostrand, 1969). Ever since, local protein synthesis has been studied in cells of the NS with a particular emphasis on neurons. To date, it is known that local translation is involved in axonal maintenance, guidance and arborization, and it promotes synapse formation and synaptic plasticity (Zhang and Poo, 2002; Martin, 2004; Yoon et al., 2012; Leal et al., 2014). Given that neuronal processes might as well receive environmental stimuli with potential detrimental effects, local protein synthesis also becomes relevant upon neuronal damage and in response to stress as previously reviewed (Baleriola and Hengst, 2015; Lin et al., 2020).

Although the local synthesis of acetylcholinesterase in axons was discovered by Koenig and Koelle (1960) it took two decades to detect the presence of ribosomes in neuronal peripheral processes: in 1982 polyribosomes (or polysomes) were detected for the first time in dendritic spines of neurons located in the dentate gyrus (Steward and Levy, 1982; Steward and Fass, 1983) and later, in 1986 polysomes were detected in axons (Steward and Ribak, 1986). The discovery of dendritic polysomes encouraged researchers to focus on mRNA localization and localized translation in dendrites, and soon various dendritic transcripts were identified, such as microtubule-associated protein 2 (*Map2a*) (Garner and Matus, 1988), calcium/calmodulin- dependent protein kinase 2 alpha (*Camk2α*) (Burgin et al., 1990) or activity-regulated cytoskeleton-associated protein (*Arc*) (Steward et al., 1998), among others. *Actb* was the first transcript identified in vertebrate axons (Bassell et al., 1998) and its translation in *Xenopus* embryos is involved in growth cone behavior during development. Other cytoskeleton- and membrane-associated proteins have more recently been identified as locally produced in rodent axons (e.g., RhoA, Par3, and SNAP25…) (Wu et al., 2005; Hengst et al., 2009; Batista et al., 2017). Transcripts encoding proteins associated with cytoskeleton and membrane dynamics are not the only locally translated in neuronal processes. Local production of proteins involved in mitochondrial function and even transcription factors have been detected in both dendrites and axons as recently reviewed by Blanco-Urrejola and colleagues (Blanco-Urrejola et al., 2021). In essence, local translation is a crucial mechanism that contributes to many neuronal functions and although this phenomenon has been mainly studied in physiological conditions it is now accepted that its deregulation is involved in various neurological diseases (Khalil et al., 2018), which we will review in this article. However, before we delve into local translation in NS pathologies, we will briefly describe the process that transcripts follow since leaving the nucleus until they get translated in target subcellular compartments.

## Local Translation: From mRNA Transport to Protein Synthesis

The canonical view of protein synthesis is that mRNA translation occurs in the soma either by ribosomes associated to the rough endoplasmic reticulum (RER) (Figure 1B^i^) or by free ribosomes present in the cytosol (Figure 1B^ii^). The newly synthesized proteins are then processed and once mature they are transported through microtubules by dyneins and kinesins to different subneuronal domains (Figure 1C^i^), where they carry out their function (Figure 1D^i^). Conversely, local protein synthesis requires transcripts to leave the nucleus and bind ribonucleoprotein (RNP) granules (Figure 1B^iii^). RNP granules are then transported to target compartments (Figures 1C^ii,iii^) and once there, mRNAs are translated into protein (Figure 1D^ii^).

### mRNA Transport to Neuronal Peripheral Processes

When mRNAs exit the nucleus, RNA binding proteins (RBPs) recognize specific localization elements typically found in the 3′-Untranslated Regions (3′-UTR) of the transcripts known as zip codes (Perry and Fainzilber, 2011; Szostak and Gebauer, 2013; Berkovits and Mayr, 2015). Interestingly, evidence suggest that long 3′-UTRs provide a longer life span to RNAs favoring their long distance transport and hence their localization to peripheral neuronal processes (Tushev et al., 2018). Besides binding the transcripts, RBPs associate with translation regulators including ribosomal proteins and other components of the translation machinery, ribosomal RNAs, and non-coding RNAs (Kim et al., 2005; Skup, 2008), and assemble into membraneless RNPs (Formicola et al., 2019; Pushpalatha and Besse, 2019; Figure 1B^iii^). RNPs bind to motor proteins directly or indirectly via adapters and the transport of the transcripts to distal compartments is initiated. Depending on the target compartment, transport occurs via microtubules bound to kinesins (in the case of axons and dendrites) (Dixit et al., 2008; Guillaud et al., 2020; Figure 1C^ii^) or through actin microfilaments associated to myosin (in axonal growth cone, presynaptic terminals and dendritic spines) (Hirokawa et al., 2010; Shirao and Gonzalez-Billault, 2013; Craig, 2018; Figure 1C^iii^). During transport, RNP granules participate in the repression of translation but they are also involved in the local translation of proteins when remodeling of the RNP granules occurs driven by external signals. Interestingly, recent evidence indicates that RNP granules bind to membranous organelles located in axons such as endosomes and mitochondria, which serve as platforms to aid with local protein synthesis (Baumann et al., 2014; Cioni et al., 2019).

### Local Protein Synthesis in Subcellular Compartments

As in the soma, the ribosomes are the molecular machines responsible for protein synthesis in subcellular domains. For many years it was assumed that ribosomes were translationally active when associated forming polysomes, as these structures where the first detected in dendrites and axons (Steward and Levy, 1982; Steward and Fass, 1983; Steward and Ribak, 1986). However, a recent study has published the existence of both polysomes and monosomes in synaptosomes. This same study suggests that while in the soma the vast majority of transcripts associate to polyribosomes, most synaptic mRNAs bind to and are translated by monosomes compared to a few synaptic transcripts that preferentially bind polysomes (Biever et al., 2020).

**FIGURE 2:**
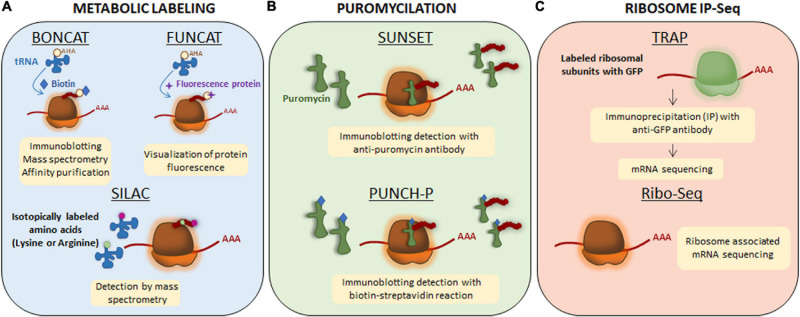
Techniques to detect newly synthesized proteins. **(A)** Metabolic labeling. Bioorthogonal non-canonical amino acid tagging (BONCAT) and fluorescent non-canonical amino acid techniques (FUNCAT) are based on non-canonical methionine derivates (an azide or an alkaline) and therefore, they are able to get incorporated to the nascent protein as methionine through endogenous methyionil-transfer RNA (tRNA) synthetase. The newly synthesized proteins will be labeled with azide or alkyne groups. For their detection, exogenous reactive groups are then incorporated to azide or alkyne groups: biotin in BONCAT and a fluorescence protein in FUNCAT. For stable isotope labeling using amino acids in cell culture (SILAC), lysine and arginine are isotopically labeled with ^12^C (light amino acids) and ^13^C (heavy amino acids). The incorporation of labeled amino acids allows them to enter into newly synthesized proteins and be detected by mass spectrometry. **(B)** Puromycilation. Puromycin is an antibiotic that structurally mimics tRNAs so it can get incorporated into the nascent polypeptide chain and elongation is stopped. Puromycilated-proteins can be detected by immunochemistry directly using an anti-puromycin antibody for surface sensing of translation technique (SUNSET) or indirectly when puromycin is bound to biotin for puromycin-associated nascent chain proteomics (PUNCH-P). **(C)** Ribosome immunoprecipitation-sequencing (IP-Seq). Translating ribosome affinity purification (TRAP) method consists on labeling ribosomal subunits with Green Fluorescence Protein (GFP) followed by immunoprecipitation approach. Transcripts bound to ribosomes containing GFP will be isolated and sequenced by RNA-sequencing. Ribo-Seq technique is based on sequencing all mRNA transcripts which are associated to ribosomes.

**FIGURE 3:**
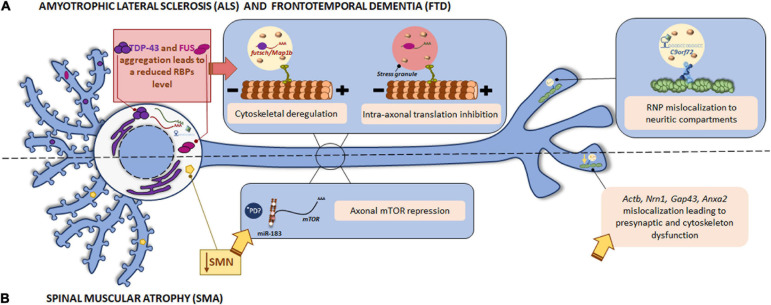
Local translation defects in ALS/FTD and SMN. **(A)** Among ALS/FTD-linked proteins, TDP-43, FUS, and C9orf72 are found. The RBPs TDP-43 and FUS play a role in RNA metabolism. The aggregates usually found in ALS and FTD patients impair TDP-43 and FUS function leading to altered mRNA localization and the consequent cytoskeletal deregulation and stress-mediated intra-axonal translation inhibition, respectively. The GGGCC repeat expansion in the *C9orf72* mRNA triggers its assembly in RNP granules, which are erroneously localized to neuritic compartments and affect to nerve branching. **(B)** Loss of SMN is the main cause of SMA. SMN is involved in the localization of mRNAs to the axonal compartment. SMN defects lead to *Actb*, *Nrn1*, *Gap43*, and *Anxa2* mislocalization with important impact in neurite growth, presynaptic function, and cytoskeleton plasticity. SMN also controls the regulator of local translation mTOR through miR-183. *PD miRNAs are also involved in the translation repression induced by mutant LRRK2 in PD. Due to local miRNAs have been identified as contributors to other pathologies, a similar local mechanism could participate in the pathogenesis of PD.

Local translation, like any other cellular process, requires energy. Recently, Rangaraju et al. (2019) described that mitochondrial compartments present in dendrites contribute to synaptic plasticity by supplying the energy required to synthesize proteins in spines. However, some subneuronal domains (e.g., the presynaptic terminals) do not contain mitochondria and the energy demands are met by local ATP produced by synaptic cycles in response to neuronal activity (Rangaraju et al., 2014). Independently on how subcellular compartments provide the necessary energy for protein synthesis, transcripts are considered locally translated if they meet the following requirements: (1) coding transcripts to be translated need to colocalize with ribosomes and other components of the translation machinery in a given subcellular domain, (2) newly synthesized proteins need to be detected at subcellular levels by techniques such as protein metabolic labeling (Figure 2A), puromycilation of nascent peptides (Figure 2B) or ribosome immunoprecipitation followed by sequencing (Figure 2C), and (3) levels of newly synthesized proteins need to drop when blocking protein synthesis (e.g., with pharmacological inhibitors such as anisomycin or emetine) (Holt et al., 2019).

## Local Translation in Nervous System Pathologies

Since the study of the first proteins synthesized in different subneuronal domains, most research has focused on discovering new localized transcripts in neurons. However, there was no evidence linking local synthesis deregulation with neuronal pathologies until 2001 when Zheng and colleagues described that intra-axonal RNA translation was activated in response to nerve injury (Zheng et al., 2001). From then on, there is growing evidence on the translation of localized transcripts upon nerve injury and on the role of locally produced proteins in the response of neurons to damage. For instance, the axonal synthesis of the transcription factors STAT3 and PPARγ after sciatic nerve crush is followed by their retrograde transport toward the neuronal cell body. Once there, both STAT3 and PPARγ trigger transcriptional programs contributing to nerve regeneration (Ben-Yaakov et al., 2012). Interestingly, at least one component of the importin α/β complex required for retrograde transport is also synthesized in injured axons (Hanz et al., 2003). These data point at the implication of local translation in neuronal survival following nerve lesions in the peripheral nervous system. In addition, a deregulation of local protein synthesis upon damage was also recently suggested in the central nervous system. In retinal ganglion cells, the RBP RBPMS is expressed exclusively in the soma but upon hypoxia and axotomy, RBPMS is redistributed to dendrites and axons, respectively (Pereiro et al., 2020). Authors have also identified alterations of various RBPs in different neurological diseases, including amyotrophic lateral sclerosis, frontotemporal dementia or spinal muscular atrophy. Likewise, in other disorders such as Alzheimer’s disease, deregulation of translation of certain mRNAs in neuronal processes seems to contribute to the pathology. We will now summarize the current knowledge on how deregulation of local protein synthesis at different levels can contribute to the development and progression of NS pathologies (Table 1).

**TABLE 1:** Summary of altered proteins and mRNAs involved in local translation in different nervous system pathologies.

**Disease**	**Protein/mRNA**	**Altered mechanism on local translation**	**Model system**	**References**
ALS/FTD	TDP-43	Abnormal trafficking of *futsch*/*Map1b* mRNAs to neurites leading to cytoskeleton defects	*Drosophila* model of ALS *in vivo*, ALS vs. control postmortem brain tissue	Coyne et al., 2014
	FUS	Stress-mediated inhibition of intra-axonal translation	HEK-293 cell line and zebrafish embryo spinal cords; mutant FUS mice and mouse hippocampal neuron culture	Bosco et al., 2010; Lopez-Erauskin et al., 2018
	C9orf72	mRNP granules mislocalization to neuritic compartments affecting to branching	Rat spinal cord neuron culture, *Drosophila* model *in vivo*	Burguete et al., 2015
SMA	SMN	*Actb*, *Nrn1*, *Gap43*, *Anxa2* mislocalization altering neurite growth, presynaptic function, and cytoskeleton Repression of axonal mTOR through miR-183	NSC-34 cell line; mouse motor neuron culture Rat hippocampal, cortical, and motor neuron culture	Rage et al., 2013; Fallini et al., 2014, 2016; Kye et al., 2014; Rihan et al., 2017
AD	*Mapt*	*Mapt* mislocalization to dendrites and hyperphosphorylation	Mouse hippocampal neuron culture, WT vs. Tau KO mice; hippocampal neuron culture from WT and Fyn KO mice, HEK-293 cell line, WT, APP23 and FynCA Tg mouse models *in vivo*	Kobayashi et al., 2017; Li and Gotz, 2017
	*Atf4*	*Atf4* recruitment to axons induced by Aβ oligomers, leading to neuronal death	Rat hippocampal neuron culture, mouse model *in vivo*, AD and control postmortem human brain tissue	Baleriola et al., 2014; Walker et al., 2018
PD	UPR genes	UPR altered. A compartment-dependent UPR involving local translation?	α-synuclein Tg mice	Hetz and Saxena, 2017
	LRRK2	Deregulation of global eIF4E/4E-BP. Defects in axonal 4E-BP dependent translation? Axonal microRNAs deregulation?	HEK-293 and SH-SY5Y cell lines; rat superior cervical ganglia neuron culture; rat cortical neuron culture, WTvs. LRRK2 KO mice	Imai et al., 2008; Kumar et al., 2010; Natera-Naranjo et al., 2010; Herzig et al., 2011; Trancikova et al., 2012
	PINK1/PARK2	Pumilio and Glorund/hnRNP-F displacement: altered translation in the mitochondrion surface (within axons?) due to *PINK1*/*PARK2* mutations.	*Drosophila* PINK1 model and HEK 293T cell line	Mouton-Liger et al., 2017; Martinez et al., 2019
HD	HTT	Impaired dendritic levels of *Actb* mRNA, Ago2 protein and P-bodies Altered axonal BDNF delivery leading to neurotoxicity	Homozygous *Htt* mutant cell lines (109Q/109Q); HeLa S3 cells Flagged-Htt590; rat cortical neuron culture and brain sections	Gauthier et al., 2004; Savas et al., 2008; Ma et al., 2010, 2011; Savas et al., 2010
ASD and FXS	FMRP	Deregulation of local mRNAs linked to abnormal spine morphology and plasticity Upregulation of dendritic *Dlg4* translation through inhibition of miR-125a with spine morphology defects Dendritic mTOR upregulation	Rat hippocampal neuron culture; cortical neuron culture from *Fmr1* KO mice; *in vivo* Fmr1 KO mouse model; Organotypic hippocampal slice cultures from *Fmr1* KO mice	Antar et al., 2004; Muddashetty et al., 2011; Pyronneau et al., 2017; Banerjee et al., 2018; Feuge et al., 2019
DS	*Dscam*	Upregulation of dendritic mRNA and protein levels with defects in dendrite branching	Mouse hippocampal neuron culture from Ts1Cje mice, DS mouse model	Alves-Sampaio et al., 2010
	*Bdnf*	Upregulation within dendrites leading to an aberrant activation of dendritic translation	*In vitro* hippocampal dendrites from Ts1Cje mice	Troca-Marin et al., 2011; Swanger and Bassell, 2013
Other	*Bdnf* (depression and bipolar disorder)	Dendritic *Bdnf* localization to dendrites disrupted	Ts1 Cje mouse hippocampal neuron culture	Swanger and Bassell, 2013
	Pumilio-2 (epilepsy)	Erroneous mRNAs localization to axons Upregulation of overall translation in axons with branching defects	*In vivo* 5 month Pum2 gene-trap mice; Pum2 knockdown in primary dorsal root ganglion rat neuron culture and mouse model *in vivo*	Follwaczny et al., 2017; Martinez et al., 2019

*ALS, amyotrophic lateral sclerosis; FTD, frontotemporal dementia; SMA, spinal muscular atrophy; AD, Alzheimer’s disease; PD, Parkinson’s disease; HD, Huntington’s disease; ASD, autism spectrum disorders; FXS, fragile-X syndrome; DS: down syndrome.*

### Amyotrophic Lateral Sclerosis (ALS) and Frontotemporal Dementia (FTD)

Amyotrophic lateral sclerosis is the most common motor neuron disease with an adult onset and it is characterized by the degeneration of upper and lower motor neurons. Most of ALS patients –around 90%– suffer the sporadic form of the disease whereas the remaining 10% of the cases correspond to familial ALS with a genetic inheritance (Scotter et al., 2015). Different genes have been associated to ALS pathology including those encoding for Cu-Zn superoxide dismutase 1 (SOD1), Tar DNA binding-protein 43 (TDP-43), fused in sarcoma/translocated in liposarcoma (FUS), or chromosome 9 open reading frame 72 (C9orf72). Interestingly, some of the ALS-linked genes have also been related to FTD. FTD is a group of disorders whose main feature is the loss of neurons in frontal and temporal brain lobes leading to cognitive impairment. Similarly to ALS, FTD is mainly a sporadic disorder although 40% of cases have family history with a 10% of autosomal dominant inheritance (Bott et al., 2014). In this section we will review the ALS/FTD-associated genes involved in mRNA localization and translation within neurites.

Tar DNA binding-protein-43 is an RBP involved in mRNA splicing, transport and translation in neuronal processes. The presence of TDP-43 aggregates in the cell cytoplasm in ALS and FTD patients has been reported. TDP-43 associates to specific mRNAs in RNP granules and transports them to the target neuronal compartment where mRNAs can be translated by the localized translation machinery (Alami et al., 2014). In 2014, *futsch* mRNA was identified as a TDP-43 target in a *Drosophila* ALS model. *Futsch* is known to be involved in the development and maintenance of synaptic contacts at the neuromuscular junctions (NMJ). Mutant TDP-43 alters *futsch* localization: mRNA levels are significantly reduced at the NMJ while higher levels are detected in neuronal somata. In addition to mislocalization, TDP-43 also regulates *futsch* translation by shifting mRNA from actively translating polysomes to non-translating ribonuclear protein particles. Similarly, the mammalian homolog of Futsch, MAP1B accumulates in motor neuron cell bodies in the spinal cord from ALS patients (Coyne et al., 2014). As both Futsch and MAP1B are part of the microtubule cytoskeleton, defects in their local translation due to mutant TDP-43 could lead to cytoskeletal defects, contributing to ALS development (as summarized in Figure 3A).

Another important RBP linked to ALS and FTD is FUS, which, as TDP-43, plays a role in RNA metabolism (splicing, trafficking and translation). FUS also appears in cytoplasmic inclusions in diseases. However, it was reported that FUS deposits are post-translationally modified by arginine unmethylation only in postmortem tissue from FTD patients but not in ALS ones. Indeed, FUS hypomethylation triggers its aggregation and consequently alters RNP granules and local protein synthesis in neurites (Qamar et al., 2018). On the other hand, wild type FUS is present in somata and along neuronal processes as well, including dendrites, spines and the NMJ. Specifically, FUS has been detected at translation sites in axons. ALS-linked mutations in FUS lead to its accumulation in neurites of hippocampal and sciatic nerve neurons. Moreover, mutant FUS leads to stress-mediated inhibition of intra-axonal protein synthesis and therefore to synaptic dysfunction (Lopez-Erauskin et al., 2018; Figure 3A). In fact, a previous study had already reported the incorporation of cytoplasmic mutant FUS into stress granules (Bosco et al., 2010). Some FUS target mRNAs are abnormally expressed when FUS is mutated. For instance, *Fos-B* is upregulated in axons in FUS-mutant motor neurons causing an increase in axonal branching. The axonal morphology can be rescued by the suppression of exacerbated *Fos-B* expression by locally applying siRNAs to axons. Interestingly, an abnormal Fos-B upregulation in ventral horn neurons in ALS autopsy samples has also been reported (Akiyama et al., 2019).

In addition to the contribution to intra-axonal translation, both FUS and TDP-43 are also localized to dendrites and dendritic spines (Swanger and Bassell, 2013). Furthermore, TDP-43 does not only regulate mRNA translation but is also involved in dendritic mRNA trafficking (Chu et al., 2019).

Notably, an interaction between FUS and TDP-43 was identified in 2010. It is of interest to highlight that this FUS/TDP-43 association is enhanced by ALS-linked mutant variants in TDP-43 (Ling et al., 2010). Therefore, this report reveals the possible convergence of pathogenic mechanisms from both FUS and TDP-43 in ALS. Similarly, other interactions between proteins associated to NS disorders have been identified. TDP-43 seems to interact to disrupted in schizophrenia 1 (DISC1) in brains of both FTD mouse model and patients. The TDP-43/DISC1 complex negatively regulates dendritic local translation in response to neuronal activity. This association might explain some psychiatric behaviors observed in FTD patients since DISC1 is a relevant player in the pathology of psychiatric disorders (Endo et al., 2018). In the case of FUS, it interacts with the survival motor neuron protein (SMN), the main responsible for the development of spinal muscular atrophy (SMA). The relationship between both disease-linked proteins will be discussed in the next paragraph corresponding to SMA.

The GGGGCC repeat expansion in the *C9orf72* gene is associated to a significant percentage of familial ALS cases. Although C9orf72 function remains unclear, authors have demonstrated that the repeat expansion can form G-quadruplexes, which are known to participate in splicing, mRNA trafficking and translation regulation (Fratta et al., 2012). In addition, GGGGCC repeat RNAs are assembled into mRNA transport granules and wrongly localized to neuritic compartments. The consequent mislocalization of mRNA granules alters nerve branching (Figure 3A). These observations suggest a novel mechanism underlying neuronal defects in disease-associated expanded repeats like ALS (Burguete et al., 2015).

As mentioned above, late endosomes act as platforms for local protein synthesis in axons and dendrites. Moreover, other organelles, including mitochondria or lysosomes, can be “vehicles” for RNP granule transport and localization. ANXA11 is an RNA granule-associated protein that acts as an adaptor between lysosomes and RNP granules. ALS-associated mutations in ANXA11 prevents RNP granule association to lysosomes and impaired RNP transport which results in altered RNA localization to axons what might contribute to axonal degeneration in ALS (Liao et al., 2019).

Together, these findings underline the relevance of a correct localization of mRNAs for neuronal physiology: while mutant forms of the RBPs TDP-43 and FUS alter localization of certain mRNAs within neurites and lead to the consequent impairment on cytoskeleton and synapses, the defective localization of mRNA granules in neurites caused by the GGGGCC repeat expansion in *C9orf72* results in altered branching (Figure 3A and Table 1).

### Spinal Muscular Atrophy (SMA)

Spinal muscular atrophy is another example of a motor neuron disease in which both RBP deregulation and mRNA mislocalization have been reported. SMA is an autosomal disorder characterized by the progressive loss of spinal motor neurons and skeletal muscle atrophy. Mutations in the *SMN1* gene leading to reduced SMN levels haven been identified as the cause of SMA pathology. SMN is a ubiquitously expressed RBP whose main function is the assembly of small nuclear RNPs for mRNA splicing. However, neuronal SMN is also involved in the assembly of RNP complexes required for mRNA transport to axons and for local translation. Fallini and colleagues described that the association of SMN to messenger RBPs (mRBPs) such as HuD and IMP1 is involved in the localization of poly(A) mRNAs to the axonal compartment (Fallini et al., 2011, 2014). Defects in axonal SMN thus lead to mRNA mislocalization. Among mislocalized mRNAs, some relevant for neurite growth and presynaptic function can be found, including *Actb*, *Nrn1*, and *Gap43*, to mention but a few. For instance, *Gap43* is reduced in growth cones of SMA motor neurons at mRNA and protein levels. Interestingly, HuD and IMP1, both of them altered by defects in SMN, control the transport of *Gap43* mRNA. Conversely, the overexpression of these mRBPs is sufficient to restore axon growth (Fallini et al., 2016). Likewise, 50% of SMN reduction impairs the localization of other mRNAs such as *Anxa2* (encoding Annexin A2) and *Cox4i2* (Rage et al., 2013). Annexin A2 is involved in actin cytoskeleton plasticity. The observed defects in cytoskeleton organization in SMN deficiency models could be explained at least in part by the mislocalization of *Anxa2* and its consequent impaired axonal translation (Rihan et al., 2017). Altogether, these data suggest that deregulation of mRNA localization to axonal compartments and local protein synthesis are key players in the development of SMA pathology.

Survival motor neuron protein also controls local translation through microRNA (miR) expression. In SMN-deficient neurons, miR-183 is increased in neurites. miR-183 targets axonal mTOR, a master regulator of local protein synthesis, leading to reduction of its own translation. Therefore, overall mTOR-dependent local translation is decreased in SMN-deficient axons (Kye et al., 2014).

We have mentioned in previous paragraphs the existent interaction between proteins associated to different neurodegenerative diseases. A 2013 study identified FUS, one of the ALS/FTD-linked RBPs, as an interactor of SMN. The association between both RBPs appears to be influenced by FUS mutations and leads to a redistribution of SMN toward FUS deposits with the consequent impairment in axonal localization of SMN (Groen et al., 2013). Moreover, there is evidence of the association of SMN and FMRP (fragile X mental retardation protein, involved in Fragile X syndrome) in neuronal mRNP granules (Piazzon et al., 2008).

To summarize, SMN contributes to local protein synthesis regulation through its interaction with RBPs and the control of miRNAs. Therefore, mutations in this protein result in mRNA mislocalization and repression of axonal translation (Table 1). The data reviewed so far overall suggest that dysfunction of RBPs and the consequent mRNA mislocalization alter localized translation and underly the pathophysiological features of motor neuron diseases as depicted in Figure 3B.

### Alzheimer’s Disease (AD)

Alzheimer’s disease is the most common cause of dementia among the elderly. It is an incurable neurodegenerative disease characterized by the gradual loss of cognitive and functional abilities. Pathological cellular dysfunction in AD begins many years before the onset of the symptoms. Two hallmarks have been identified in postmortem brains as main contributors to AD pathology: the neurofibrillary tangles, composed of hyperphosphorylated microtubule associated protein Tau (MAPT or Tau) and the extracellular β-amyloid (Aβ) plaques (Tiwari et al., 2019; Busche and Hyman, 2020). Although the mechanisms leading to the development and spread of the disease through the brain are not fully understood, it is known that synapse loss is one of the first pathological signs of AD (Selkoe, 2002). Alterations in the axonal and dendritic proteomes could underlie synaptic dysfunction and thereby the study of local protein synthesis in this context might provide clearer knowledge on the development of the disease.

Tau is mainly localized to axons though is also present in the somatodendritic compartment at very low levels in healthy neurons (Tashiro et al., 1997). Axonal Tau is partially the product of locally translated *Mapt* mRNA (Aronov et al., 2001; Gauthier-Kemper et al., 2018). Nevertheless, *Mapt* gets aberrantly localized to dendrites in tauopathies such as AD. For instance, Aβ peptides induce *Mapt* mRNA localization to dendrites and its localized translation. Intriguingly, the mislocalization and dendritic synthesis of Tau enhances its hyperphosphorylation (Kobayashi et al., 2017; Li and Gotz, 2017), indicating that Tau synthesis in the inappropriate subneuronal compartment contributes to neurodegenerative diseases such as AD.

**FIGURE 4:**
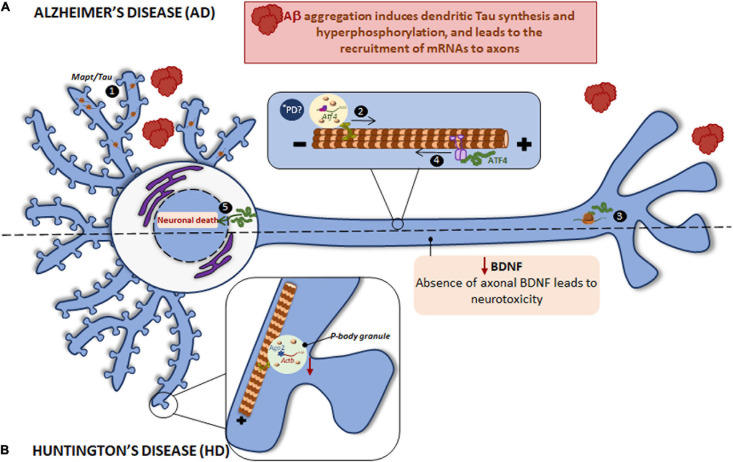
Local translation defects in AD and HD. **(A)** The two main hallmarks in AD brains are the accumulation of intracellular hyperphosphorylated Tau and extracellular Aβ plaques. Aβ induces the incorrect localization of *Mapt* mRNA to dendrites with the consequent, local synthesis of Tau and its hyperphosphorylation (1). Aβ also induces the recruitment of certain mRNAs to axons, among them *Atf4* is found (2). Once ATF4 is axonally synthesized (3), it is retrogradely transported to cell body (4) where enhances death transcriptional programs (5). *PD ATF4 is also a component of UPR. The UPR is altered in PD and therefore the local ATF4 could be involved in PD progression. **(B)** HD is caused by a mutant CAG expansion in *HTT* gene. HTT KO leads to reduced levels of *Actb* mRNA, Ago2 protein, and p-bodies in dendrites. Mutant HTT impairs BDNF transport to axons resulting in neurotoxicity and probably affects to local protein synthesis since BDNF regulate this process in subneuronal compartments.

Aβ peptides are the result of incorrect proteolytic cleavage of amyloid precursor protein (APP) and are prone to aggregate, which results in the formation of senile plaques (Rabbito et al., 2020). We have stated that Aβ triggers Tau synthesis in dendrites but it also induces local protein synthesis in axons (Baleriola et al., 2014; Gamarra et al., 2020). The mRNA encoding the transcription factor ATF4 (previously known as CREB2) is among the proteins whose local translation is induced by Aβ peptides. Interestingly, axonally synthesized ATF4 is retrogradely transported to the nucleus where it alters transcription leading to neuronal death both *in vitro* and *in vivo* (Baleriola et al., 2014; Figure 4A). Additionally, *Atf4* recruitment to Aβ-exposed axons is itself triggered by local translation of sentinel mRNAs such as the intermediate filament protein vimentin (*Vim*) (Walker et al., 2018). These studies suggest a common axon-to-soma communication mechanism in injured nerves and in response to amyloid based on axonally synthesized transcription factors although the outcome of the transcriptional response differs: a regenerative response of injured nerves vs. neuronal death following the local exposure to Aβ oligomers.

*Atf4* is not the only mRNA found in Aβ-treated axons. Interestingly, transcripts from 9 of the 20 susceptibility genes associated to late onset AD are axonally localized following exposure to Aβ oligomers: *App, Clu, ApoE, Sorl1, Bin1, Picalm, Ptk2, Celf1*, and *Fermt2* (Blanco-Urrejola et al., 2021).

Local translation has also been described in other neural cells like oligodendrocytes. For example, the heterogeneous nuclear RNP A2 (hnRNP A2) is part of the transport granules responsible for mRNA localization to peripheral processes in oligodendrocytes (Hoek et al., 1998; Munro et al., 1999). Intriguingly, loss of hnRNP A/B in entorhinal cortices from AD brains has been reported. Experiments performed in neurons indicate that defects in hnRNP A/B lead to dendritic loss *in vitro* and memory and electrocorticographic alterations *in vivo* (Berson et al., 2012). Based on the role of hnRNP A2 in oligodendrocytes a possible involvement of hnRNP A/B in neuronal mRNA metabolism cannot be discarded. Further investigation should be carried out in order to fully understand the role of hnRNPs in RNA localization and protein synthesis in neuronal processes in disease.

Regarding interaction between proteins associated to different NS pathologies, in 50% of FTD cases an accumulation of Tau fibrils has been observed (Goedert et al., 2012). Interestingly, reduced levels of the ALS/FTD-linked TDP-43 are detected in the AD brain. Indeed, TDP-43 promotes *Mapt* mRNA instability through its 3′-UTR, suggesting that altered TDP-43 might contribute to Tau aggregation (Gu et al., 2017). Taking into account that axonal localization signals are usually located at the 3′-UTR, we can speculate that deregulation of TDP-43 also affects Tau localization and therefore might also contribute to Tau pathology in FTD.

Results reviewed in these paragraphs strongly point toward the importance of mRNA localization and localized translation and/or deregulation of both phenomena in the development of AD and related disorders. One of the main hallmark of AD, Aβ aggregates, lead to deregulation of local protein synthesis in both dendrites and axons. These events contribute to Tau hyperphosphorylation and neuronal death respectively (Table 1). Thus, restoring levels of localized mRNAs and/or their localized translation could alleviate cell dysfunction linked to AD.

### Huntington’s Disease (HD)

Huntingtin (HTT) is a protein expressed at high levels in several brain regions like the hippocampus, the cortex, the cerebellum and the striatum. CAG repeat expansions in the *HTT* gene result in a repeated polyQ tract in the N-terminal region of the HTT protein, causing HD. HD is an autosomal dominant neurodegenerative disease characterized by uncontrolled movements, and behavior and cognitive impairments (Landles and Bates, 2004; Li and Li, 2004). HTT, as well as APP, is transported along axons and has been implicated in dendritic RNA delivery (Ma et al., 2010; Savas et al., 2010). Importantly, HTT inactivation leads to impaired localization of RNAs and membranous organelles (Gauthier et al., 2004), indicating that HTT plays an important role in RNA transport.

Similar to other previously mentioned neurodegenerative disorders, RBPs colocalize with the main driver of HD: HTT interacts with Ago2 and Staufen in processing bodies (P bodies) which contain translationally repressed mRNAs (Savas et al., 2008), and in dendritic RNA granules involved in transport and local translation (Savas et al., 2010). Additionally, HTT was reported to bind to the 3′-UTR of dendritically targeted mRNAs including *Ip3r1*, *Actb*, and *Bdnf*. Moreover, HTT knockout (KO) reduces levels of dendritic *Actb* mRNA, Ago2 protein, and P bodies (Ma et al., 2010, 2011; Savas et al., 2010). HTT is also required for BDNF vesicular transport along microtubules toward the axons. Mutant HTT impairs BDNF transport leading to neurotoxicity (Gauthier et al., 2004) (as summarized in Figure 4B). Noteworthy, BDNF is known to regulate local translation (Swanger and Bassell, 2013) and thus local protein synthesis in HD might be impaired via defective axonal BDNF delivery.

Huntingtin is thus yet another example of a main contributor to NS pathology involved in mRNA trafficking to neuronal processes by its interaction with RBPs or by its direct binding to mRNA 3′-UTRs (Table 1).

### Parkinson’s Disease (PD)

Parkinson’s disease is a chronic neurodegenerative disorder in which dopaminergic neurons of the *substantia nigra* progressively die and there is an accumulation of Lewy bodies composed of abnormal intracellular aggregates of alpha synuclein protein (αSyn) and ubiquitin (Goedert et al., 2013). The pathogenic process in PD involves regions of the central and peripheral NS with bradykinesia, rigidity and other motor symptoms being the main clinical manifestations (Halliday and McCann, 2010). As in AD, pathological signs at cellular levels appear before clinical symptoms and lead to the loss of 60-70% of neurons in the *substantia nigra pars compacta* (Radhakrishnan and Goyal, 2018).

Impairment of local protein synthesis in PD has not been fully demonstrated. However, a recent review by Lin and colleagues commented on this possibility. Given their complex morphology, neurons cope with stress in a compartmentalized manner (Lin et al., 2020). In PD, as in AD, prion disease and other disorders in which protein aggregation occurs, stress responses in neurons are evident before the pathological hallmarks of the disease are apparent. Oxidative stress and ER stress induce mechanisms able to cope with basal levels of neuronal damage. However, when a certain threshold is reached, those same mechanisms might backfire and contribute to neurodegeneration. Interestingly, the dual role and compartmentalized nature of stress mechanisms was implicitly reported in a mouse model of acute amyloid pathology where locally translated ATF4 mediates neurodegeneration while global increases of this same transcription factor allows neurons to adapt to Aβ-induced stress (Baleriola et al., 2014). ATF4 is an important component of the unfolded protein response (UPR). The UPR involves a global shutdown of protein synthesis via eIf2α while promoting translation of mRNAs encoding stress-related proteins. ATF4 is classically synthesized upon accumulation of unfolded and misfolded proteins and the consequent UPR activation due to ER stress. However, in Aβ-treated axons, ATF4 translation (likely via eIf2α) but no other canonical signs of the UPR, such as protein synthesis shutdown, are apparent. A partial, non-canonical activation of the UPR has also been observed in developing axons when challenged with Sema3a (Cagnetta et al., 2019). The UPR is known to be altered in AD, PD with Lewy bodies, prion disease, HD and even ALS (Hetz and Saxena, 2017). It would be interesting to address if in neurodegenerative diseases other than AD, such as PD, a compartment-dependent UPR involving local translation contributes to disease progression (Figure 4A).

On the other hand, several studies implicate translation in PD pathogenesis. Proteins encoded by PD-associated genes have recently been shown to interact with components of the translation initiation complex. For instance, Leucine-rich repeat kinase 2 (LRRK2) whose mutations are involved in autosomal dominant Parkinson’s disease, phosphorylates 4E-BP, releasing the repression from the initiation factors eIF4E/eIF4G. Mutant forms of LRRK2 in *Drosophila* stimulate eIF4E inducing aberrantly high protein synthesis accompanied by loss of dopaminergic neurons (Imai et al., 2008). LRRK2 is also known to regulate eIF4E/4E-BP in mammals but results seem to be conflicting. Whereas *in vitro* experiments indicate increased 4E-BP phosphorylation induced by LRRK2 (Kumar et al., 2010), Trancikova and colleagues did not observe any effect on 4E-BP with *in vivo* gain- or loss-of-function approaches (Trancikova et al., 2012). Others, however, have observed increased phospho-4E-BP levels in LRRK2 KO and LRRK2 kinase dead but not in knock-in transgenic mice (Herzig et al., 2011). The so far inconclusive results on the effect of LRRK2 on the eIF4E/4E-BP axis might be due to the analyses being performed in whole cells and tissues. It would be interesting to determine whether in PD, as in Aβ pathology, 4E-BP dependent translation is enhanced in axons (Baleriola et al., 2014).

Work from Gehrke and colleagues suggested that translation repression induced by pathogenic LRRK2 is elicited through repression of miRNAs. Interestingly, miRNAs can be found in axons (Natera-Naranjo et al., 2010) and as mentioned before miR-183 regulates axonally synthesized mTOR which in turn modulates local protein synthesis in SMA (Kye et al., 2014). It would be worth determining if a similar local mechanism is involved in the pathogenesis of PD (see ^∗^PD in Figure 3B).

**FIGURE 5:**
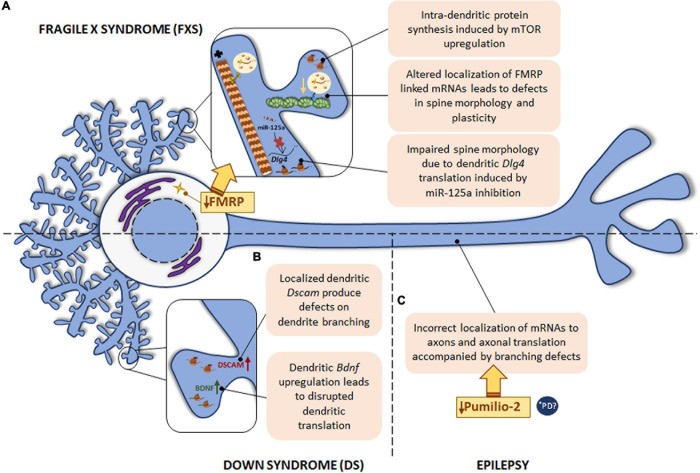
Local translation defects in FXS, DS and epilepsy. **(A)** The most common monogenic cause of ASD is FXS, caused by a mutation in *FMR1* gene with the consequent FMRP protein deficiency. FMRP regulates RNA metabolism at different levels and therefore, intra-dendritic translation is altered at several points: mTOR-mediated local protein synthesis is upregulated; deregulation of local mRNAs-linked to FMRP leads to abnormal spine morphology and plasticity; deregulation of miR-125a induces *Dlg4* mRNA dendritic translation with spine morphology defects. **(B)** DS, caused by trisomy of chromosome 21, is characterized by defects in dendrite morphology, which can be due to the increased levels of *Dscam* and *Bdnf* mRNAs in dendrites. The localization of *Bdnf* to dendrites is also disrupted in psychiatric disorders. **(C)** In epilepsy, deficiency in the RBP Pumilio-2 results in incorrect localization of mRNAs to axons and increased overall intra-axonal translation with the consequent branching defects. *PD PINK1 and PARK2, involved in PD pathogenesis, regulate translation of mitochondrial proteins by displacement of Pumilio-2 and other RBPs. Due to the local role of Pumilio-2 in translation in developing axons, localized translation in PD could also be altered.

Mutations in Pten-induced kinase 1 (PINK1/PARK6) or Parkin (PARK2), which are involved in early-onset autosomal recessive PD alter mitochondrial function. PINK1 and PARK2 promote mitochondrial localization and translation of specific mRNAs at the mitochondrial outer membrane (Gehrke et al., 2015). Translation activation via PINK1 and PARK2 implies the displacement of the repressive RBPs Pumilio and Glorund/hnRNP-F (Mouton-Liger et al., 2017). Both proteins influence mitochondrial function and play an important role in translation. Whether both RBPs play a role in localized translation in PD remains unknown. Interestingly, however, Pumilio 2 is known to regulate the local transcriptome of developing axons (Martinez et al., 2019) (^∗^PD in Figure 5C).

Although there are so far no concluding remarks on how local translation could contribute to PD pathogenesis, the fact that global protein synthesis is likely aberrantly high and that there are similarities with AD and HD strongly point to the possibility that RNA localization and localized translation in neurons are compromised in this disease and might even contribute to its progression.

### Autism Spectrum Disorders (ASD) and Fragile X Syndrome (FXS)

Autism spectrum disorders is a term referred to heterogeneous behavioral disorders with a combination of difficulties in different areas including intellectual, communication or social interaction. The most common monogenic cause of ASD is FXS, a neurodevelopmental disorder which causes intellectual disability attributed to FMRP protein deficiency as a result of a mutation in the *FMR1* gene (Salcedo-Arellano et al., 2020). One of the main cell-pathological features of FXS is the high levels of dendritic spines with an abnormal morphology linked to delayed maturation (Banerjee et al., 2018).

Fragile X mental retardation protein is a well-known RBP whose implication in dendritic local translation has been widely studied. FMRP is involved in several steps of local protein synthesis: from mRNA trafficking to peripheral processes to ribosome stalling or microRNA regulation. In relation to mRNA transport, Feuge et al. (2019) described that FMRP binds to *Cof1* mRNA and regulates its localization to and translation within dendrites. *Cof1* encodes cofilin 1, an actin-binding protein involved in dendritic spine structure and plasticity, and it was recently associated with the FXS pathology (Pyronneau et al., 2017). In *Fmr1* KO mice, defects on local *Cof1* mRNA were observed with the consequent plasticity impairment (Feuge et al., 2019). Additionally, FMRP target mRNAs encoding synaptic proteins have also been reported, including *Arc*, *Camk2a*, *Dlg4*, or *Map1b*. Interestingly, *Frm1* mRNA itself is localized to dendrites (Antar et al., 2004; Banerjee et al., 2018). Hence, the loss of FMRP leads to mislocalization and altered local translation of FMRP binding mRNAs, and FMRP deficiency is likely linked to defects in spine morphology and in neuronal plasticity observed in FXS.

Besides interacting with target mRNAs, FMRP can also bind reversibly to ribosomes and paralyzes the elongation phase of translation (Feng et al., 1997; Darnell et al., 2011; Chen et al., 2014). Furthermore, deregulated miRNAs were observed in postmortem brain tissue from ASD patients (Abu-Elneel et al., 2008). Interestingly, PSD-95 (encoded by *Dlg4*) local synthesis in dendritic compartments is mediated by the also dendritically localized miR-125a in FXS. FMRP is required for miR-125a activity through the RNA-induced silencing complex (RISC). Indeed, in *Fmr1* KO neurons *Dlg4* translation is increased while miR-125a levels are reduced in *Fmr1* KO synaptoneurosomes, affecting spine morphology and branching (Muddashetty et al., 2011).

Fragile X mental retardation protein binds ALS/FTD-linked TDP-43 to regulate the transport of mRNP granules in mouse dendrites. The association between FMRP and TDP-43 RBPs in mRNPs trafficking again opens new venues to shed light on common pathophysiological mechanisms of mRNA localization and localized translation underlying neurodevelopmental and neurodegenerative diseases (Chu et al., 2019).

Despite most evidence links FMRP to local translation in dendrites, FMRP-containing RNP granules have been detected in developing and mature axons as well (Christie et al., 2009; Akins et al., 2012, 2017). Additionally, axonal *Map1b* and *Calm1* mRNAs have been identified as miR-181d targets. FMRP appears to associate with miR-181d and its target mRNAs in dorsal root ganglion neurons (DRGs) upon nerve growth factor (NGF) stimulation and regulates axon outgrowth. FMRP deficiency *in vitro* and in a FXS mouse model leads to the mislocalization of miR-181d, *Map1b* and *Calm1* (Wang et al., 2015). Further research is required on this regard to clarify the involvement of FMRP in intra-axonal protein synthesis and the more than likely contribution thereof to FXS pathology.

Finally, FMRP is able to regulate mTOR activity in dendrites. mTOR is not only considered a regulator of axonal translation but it is also involved in intra-dendritic protein synthesis. In *Fmr1* KO mice and in studies of FXS patients, an upregulation of mTOR was detected. In addition, mTOR is also altered in other FMRP-deficiency diseases, such as tuberous sclerosis, Rett syndrome, and Down syndrome (Hagerman et al., 2010; Wang et al., 2010; Troca-Marin et al., 2011).

From all the documented evidence gathered in this section it seems clear that FXS is one of the best examples of the extent to which deregulation of local mRNA translation contributes to disease progression. The abnormal morphology of dendritic spines typically observed in FXS could be explained by the wide involvement of FMRP in dendritic local protein synthesis. As summarized in Table 1, FMRP defects entail deregulation of dendritically localized mRNAs and miRNAs, which results in spine morphology and plasticity impairment. Furthermore, dendritic mTOR is also controlled by FMRP. All this knowledge will allow a better understanding of ASD and will hopefully help develop new therapeutic strategies (Figure 5A).

### Down Syndrome (DS)

Down syndrome is the most common genetic cause of intellectual disability triggered by trisomy of human chromosome 21. Neurological symptoms are accompanied by abnormal physical growth. In some DS cases congenital heart defects are apparent. Duodenal stenosis or leukemia are also more frequent in DS patients than in the general population (Antonarakis et al., 2004). Although the specific mechanisms leading to neurological signs remain poorly understood, it is known that DS is characterized by defects in dendrite morphology and synaptic plasticity similarly to other developmental diseases like FXS (Alves-Sampaio et al., 2010). The mRNA encoding the DS cell adhesion molecule (DSCAM) is present in dendrites and DSCAM has been suggested to play a role in synaptic plasticity. Therefore, altered *Dscam* mRNA localization and defects in DSCAM local synthesis might contribute to DS-related pathogenic features in neurons. In fact, *Dscam* mRNA levels as well as its localized translation are increased within dendrites of hippocampal neurons leading to a negative effect on dendrite branching in a DS mouse model (Alves-Sampaio et al., 2010). Additionally, the same DS model presents increased dendritic levels of BDNF, which is a local protein synthesis modulator (Swanger and Bassell, 2013). Consequently, the rate of local translation in dendrites is aberrantly increased (Troca-Marin et al., 2011; Figure 5B).

### Other Neurological Disorders

Interestingly, a point mutation in the human *BDNF* gene is associated with depression and bipolar disorder. The presence of the human *BDNF* mutation in a mouse model disrupts the localization of *Bdnf* mRNA to dendrites (Swanger and Bassell, 2013). These data suggest that altered *Bdnf* localization and the consequent defects in its translation might contribute to psychiatric disorders (Figure 5B), although this contribution should be explored further.

Finally, epilepsy is also associated with deficiencies in RBPs. For instance, in 2017 Pumilio-2 was described as an RBP involved in epileptogenesis (Follwaczny et al., 2017). A recent study has described Pumilio-2 as a regulator of the axonal transcriptome in developing axons, whose mechanism of action is the exclusion of RNAs from the axonal compartment by retaining them in the cell body. Importantly, Pumilio-2 knockdown not only leads to the erroneous axonal mRNAs localization and increased overall translation levels in axons, but also to branching defects (Martinez et al., 2019) as summarized in Figure 5C.

To sum up, increasing evidence suggest that mRNA localization and local protein synthesis might be a key contributor to NS pathological conditions, not only in neurodegenerative diseases but also in other neurological disorders as discussed in these paragraphs.

## Concluding Remarks

The data reviewed in this article bring to light the increasing body of evidence on the relevance of mRNA localization and local protein synthesis not only in brain development and physiology but also in NS pathologies including neurodegenerative diseases (Table 1). We cannot, however, overlook the limitations in studying local protein synthesis. While we can separate different subneuronal compartments *in vitro* using diverse specialized culture systems (microfluidic chambers, modified Boyden chambers…), this task cannot be performed *in vivo*. In whole animals, neuronal processes are intermingled and cannot be found in isolation. Even when synaptosomes can be isolated from entire tissues, thorough controls have to be performed in order to ensure that RNAs and proteins of interest are indeed localized to neuronal peripheral domains and/or are a result of local protein synthesis. Additionally, often times the amount of material obtained from synaptosomes or brain regions enriched in neurites (e.g., the neuropil) is limiting. Identification of specific transcripts and proteins along neuronal processes is possible also by *in situ* hybridization and immunohistochemical approaches, which can also be performed in human brain tissue and thereby, this kind of samples contribute to clarify the findings in culture and animal brains, supporting the significance of localized mRNAs and the translation machinery in several pathophysiological contexts. However, postmortem tissue does not allow mechanistic assessment and cell-compartment isolation from brain neurons is not possible at clinical stages. Human studies are thus one of the main challenges in the field of local protein synthesis.

On the other hand, localized translation research in the context of pathologies provides new exciting venues for the development of novel therapeutic targets for nervous system pathologies although localized strategies should be explored more in detail. For instance, current technologies do not allow the knockdown of specific axonal mRNAs without altering the somatic counterparts. Every effort should be done to develop new approaches on this matter. Nevertheless, it is worth noting that local protein synthesis in neurons does not seem to rely only on the neurons but that glial cells might contribute to this phenomenon. Interestingly, in the peripheral nervous system, Schwann cells transfer ribosomes to injured sciatic nerves (Court et al., 2008) whereas in the spinal cord, a transference of ribosome-like particles at the axonal-myelin sheath interface has suggested (Li et al., 2005). These data point at the possibility of a novel therapeutic strategy based on modulating glia-neuron transference instead of interfering directly with translation at subneuronal compartments. In either case, further research is still required.

## Author Contributions

JB conceived the work. MG, AC, and MB-U drafted the manuscript. MG, AC, and JB composed figures and tables. MG, AC, MB-U, and JB performed literature searching and edited the manuscript. All authors contributed to the article and approved the submitted version.

## Conflict of Interest

The authors declare that the research was conducted in the absence of any commercial or financial relationships that could be construed as a potential conflict of interest.
